# Age and sex-specific disability-free life expectancy in urban and rural settings of Bangladesh

**DOI:** 10.1186/s12963-024-00327-z

**Published:** 2024-04-20

**Authors:** Khandaker Tanveer Ahmed, Aziza Afrin, Mehedi Hasan, Sajjad Bin Sogir, Labiba Rahman, Md. Karimuzzaman, Kazi Arifur Rahman, Md. Moyazzem Hossain, Hafiz T. A. Khan

**Affiliations:** 1 Department of Statistics and Data Science, Jahangirnagar University, Savar, Dhaka, 1342 Bangladesh; 2 DREXEL Dornsife School of Public Health, DREXEL University, Philadelphia, PA USA; 3 Office of the Deputy Commissioner, People’s Republic of Bangladesh, Satkhira, Bangladesh; 4 School of Mathematics, Statistics and Physics, Newcastle University, Newcastle Upon Tyne, Tyne and Wear NE1 7RU UK; 5 Public Health & Statistics, College of Nursing, Midwifery and Healthcare, University of West London, Brentford, UK

**Keywords:** Disability-free life expectancy, Sullivan method, Sex-specific, Bangladesh

## Abstract

**Background:**

Disability-free life expectancy (DFLE) has been used to gain a better understanding of the population’s quality of life.

**Objectives:**

The authors aimed to estimate age and sex-specific disability-free life expectancy (DFLE) for urban and rural areas of Bangladesh, as well as to investigate the differences in DFLE between males and females of urban and rural areas.

**Methods:**

Data from the Bangladesh Sample Vital Statistics-2016 and the Bangladesh Household Income and Expenditure Survey (HIES)-2016 were used to calculate the disability-free life expectancy (DFLE) of urban and rural males and females in Bangladesh in 2016. The DFLE was calculated using the Sullivan method.

**Results:**

With only a few exceptions, rural areas have higher mortality and disability rates than urban areas. For both males and females, statistically significant differences in DFLE were reported between urban and rural areas between the ages of birth and 39 years. In comparison to rural males and females, urban males and females had a longer life expectancy (LE), a longer disability-free life expectancy, and a higher share of life without disability.

**Conclusion:**

This study illuminates stark urban–rural disparities in LE and DFLE, especially among individuals aged < 1–39 years. Gender dynamics reveal longer life expectancy but shorter disability-free life expectancy for Bangladeshi women compared to men, emphasizing the need for targeted interventions to address these pronounced health inequalities.

## Introduction

Life expectancy, once a sufficient measure of population health, should now be complemented with the indicator Disability-Free Life Expectancy (DFLE) to capture both quantity and quality of life, reflecting a shift from longevity to a holistic understanding of well-being [15, 28]. Life Expectancy (LE) measures expected lifespan without indicating quality of life. Disability-Free Life Expectancy (DFLE) improves understanding, while health expectancy, assessing various health indicators, serves as a valuable measure for comparing different groups [15, 24, 29].

On a global scale, the critical issue of population aging stems from the interplay of increasing LE and declining fertility, with developed countries experiencing a faster growth in the elderly population compared to the younger demographic [1, 8, 9, 11, 17, 18, 21, 34, 39]. For instance, from 1986 to 2004, Japan experienced a reported reduction in disability until 1995, after which there was an expansion in morbidity [39]. Various factors, including age, gender, urbanicity, per capita GDP, and healthcare accessibility, significantly contribute to health disparities globally, with a more pronounced impact in developing countries [1, 9, 20, 21]. Exploring age and gender disparities, research indicates that although women generally live longer, those aged 60 and above may experience higher disability prevalence and shorter Disability-Free Life Expectancy (DFLE) compared to their male counterparts, underscoring the nuanced dynamics of health outcomes [1].

Bangladesh, the world’s 8th most densely populated country with 158.90 million people, demands increased focus on the well-being of its elderly population and disability-oriented research initiatives, despite being underexplored in previous studies [3, 16, 20, 34, 40, 41]. Notably, a prior survey in 2010 revealed female and male life expectancies at age 60 to be 17.95 and 16.87 years, respectively, indicating a crucial need for contemporary insights [2, 34]. While a study [34] highlighted improvements in work-loss days for males aged 15–54 from 2004 to 2007, another study [15] concurrently revealed significant urban–rural disparities, including higher rural mortality and disability rates and distinct gender-specific patterns. Our research further contributes by conducting a nuanced comparison of life expectancy and Disability-Free Life Expectancy (DFLE) across diverse age groups, utilizing the latest datasets from HIES 2016 and Bangladesh Sample Vital Statistics 2016. This study seeks to address a significant update in the existing literature by scrutinizing age and sex-specific disability-free life expectancy in both urban and rural settings, utilizing the latest available data to offer a comprehensive insight into the intricate health dynamics of Bangladesh.

## Materials and methods

### Data collection

Because age-sex-specific life expectancies for urban and rural regions were not found for 2016, these were computed utilizing the age-sex-specific mortality rate of urban and rural areas from Bangladesh Sample Vital Statistics 2016 report (Statistics 2018). By integrating the period life tables to compute age and sex-specific disability-free life expectancy (DFLE) for urban and rural areas, age-sex-specific disability prevalence by urban and rural areas was derived from Bangladesh’s Household Income and Expenditure Survey (HIES)-2016 [4], addressing the same population as Bangladesh Sample Vital Statistics 2016’s SVRS dataset, with a similar definition of urban and rural areas using the country’s own geographic and locality codes in the enumeration areas, as sampled using similar Primary Sampling Units (PSU) chosen from a list of Housing and Population Census 2011’s enumeration areas.

Traditional life table notations are extensively used in this work. The observed mortality rate, _*n*_*m*_*x*_, from the Sample Vital Registration System 2016, a dataset officially published by the Bangladesh Bureau of Statistics (BBS) [3], was converted into the probabilities of dying, _*n*_*q*_*x*_ Bangladesh’s only national statistical office, the Bangladesh Bureau of Statistics (BBS), has released the Sample Vital Registration System (SVRS) 2016. Although, it has been studied that vital registration for death and birth in Bangladesh in 2016 wasn’t collected very accurately [33], but this study used SVRS 2016 data which is of high quality and collected through a nationwide survey carried out by the BBS under a special project. The SVRS data have been assessed for the quality of age data, which is crucial for predicting the majority of critical rates and ratios. In order to achieve this, three well-known indices: Myer’s index, Whipple’s index, and UN Age-Sex Accuracy Index have been calculated from reported age distributions by sex. Such indices have demonstrated that age reporting in SVRS 2016 has improved over the past years [3]. It is in charge of gathering, compiling, and disseminating data on vital statistics and population-related issues. The following formula was used to determine the likelihood of death:

$${}_{n}{q}_{x}= \frac{n {. }_{n}{m}_{x}}{1+ \left(n-{ }_{n}{a}_{x}\right) { }_{n}{m}_{x}}$$

where, $n$ is the length of the age group, and ${}_{n}{a}_{x}$ is the average number of person-years lived by those dying in the interval from $x$ to $x + n$. This study used age interval of 5 years for the age groups. Due to a shortage of ${}_{n}{a}_{x}$ for Bangladesh in 2016, Swedish 1952 ${}_{n}{a}_{x}$ from the Human Mortality Database [19] was used to translate from ${}_{n}{m}_{x}$ to ${}_{n}{q}_{x}$. Researchers [25] stated some strategies for choosing values of ${}_{n}{a}_{x}$, where they maintained that if no values for ${}_{n}{a}_{x}$ is available for a certain population, or a similar set of values is not found in other populations, then a simple and agreeable approach is to adopt those values. The borrowed values should be acceptable for the gender for which they are being utilized because ${}_{n}{a}_{x}$ values vary substantially between the genders. A similar method was adopted in a similar kind of study as this study [15] where Swedish 1945 ${}_{n}{a}_{x}$ was used. According to the methods of picking ${}_{n}{a}_{x}$ [36] [25], the Swedish 1952 ${}_{n}{a}_{x}$ were deemed adequate for computing period life tables for Bangladesh in 2016, after thoroughly checking for matches between the two population’s ${}_{n}{a}_{x}$ values for the gender for which they are being utilized, and the age interval wise data by gender of Bangladesh’s data matched the most with Swedish 1952 data. The set of Swedish 1952 ${}_{n}{a}_{x}$ from the male life table was used to compute both urban and rural male life tables for Bangladesh for 2016, while the set of Swedish 1952 ${}_{n}{a}_{x}$ from the female life table was used to compute both urban and rural female life tables for Bangladesh. Finally, using the set of determined ${}_{n}{q}_{x}$ and the normal processes for period life table computations as described in Demography: Measuring and Modeling Population Processes [36], sex-specific period life tables for Bangladesh in 2016 for urban and rural areas were computed.

### Estimation disability prevalence

In assessing age-sex-specific disabilities in urban and rural areas, the HIES-2016, formulated by the BBS [4], served as the primary data source. This comprehensive survey encompassed family income, expenditure, education, employment, health, and disability information. Employing a stratified, two-stage sample design, 2304 Primary Sampling Units (PSU) were selected from 2011 Housing and Population Census enumeration areas, with 20 houses per PSU chosen for interviews. The final sample included 46,080 households, with 186,055 individuals aged 5 years and above interviewed between April 1, 2016, and March 31, 2017. Stratification occurred at the district level, resulting in 132 sub-strata, comprising 64 urban, 64 rural, and four city corporations. Utilizing the country’s geographic and locality codes, the urban–rural classification was established. Implicit stratification by month was incorporated, and interviewers inputted daily data into laptops, revisiting homes for necessary modifications if inconsistencies or missing data were identified [4].

The HIES-2016 seamlessly integrated the Washington Group’s six disability-related items into a comprehensive questionnaire covering household aspects such as income, expenditure, education, employment, health, etc. The construction of these questions was guided by the World Health Organization’s International Classification of Functioning, Disability, and Health [21], providing a concise conceptual framework for understanding disability. The six disabilities were: (a) vision; (b) hearing; (c) walking and climbing; (d) difficulty in remembering and concentrating; (e) self-care; and (f) speaking and communicating. To assess these disabilities, every member of the household was asked the following questions: (a) Does (name) have difficulty seeing, even if he or she is wearing glasses? (b) Does (name) have difficulty hearing, even if he/ she is wearing a hearing aid? (c) Does (name) have difficulty walking or climbing steps? (d) Does (name) have difficulty remembering or concentrating? (e) Does (name) have difficulty with self-care, such as washing all over or dressing, feeding, or toilet chores? and (f) Does (name) have difficulty communicating, for example, understanding or being understood?

Each question had four response categories: no difficulty; yes, some difficulty; yes, severe difficulty; yes, can’t see/hear/walk/remember/self-care/communicate at all. All these were categorized into two groups as no (no difficulty), indicating no disability, and yes (yes, some difficulty/yes, severe difficulty/yes, can’t see, hear, walk, remember, self-care, communicate at all), indicating disability. Ultimately, the six forms of disability were integrated into one measure to assess the prevalence of disability, which was defined as having at least one disability. Individuals with at least one of the six disabilities were declared disabled. This approach of assessing impairment was previously applied with HIES-2010 data in Bangladesh [15], and a similar strategy had previously been used in several researches in other countries [22, 23, 37] and in some studies of Bangladesh [34, 35].

### Sampling weights of HIES 2016

For each sampling stage and each PSU within a District (Zila), the sampling probability was determined differently. In the case of HIES 2016, a two-stage, stratified clustered design, the likelihood of being selected for the sample depends on two factors: (1) the likelihood that a PSU will be selected in the first stage, and (2) the likelihood that a household within each PSU will be selected in the next stage. The formula below can be used to calculate this,

$${p}_{hij}= {p}_{1}* {p}_{2}= \frac{{k}_{h}{n}_{hi}}{{N}_{h}}* \frac{{m}_{hi}}{{n{\prime}}_{hi}}$$

where ${p}_{hij}$ is the likelihood that household $j$, in stratum $h$, and PSU $i$ will be represented in the sample. The likelihood that the PSU will be chosen in the first step is expressed as ${p}_{1}$ and ${p}_{2}$ is the likelihood that a household would be chosen in the second stage; ${k}_{h}$ is the number of PSUs chosen in stratum $h$; ${m}_{hi}$ is the number of PSU $hi$ households that were chosen, while ${N}_{h}$ is the total number of households in stratum $h$ (Statistics 2018).

### Estimating DFLE

To merge the estimated life tables and disability prevalence from the above mentioned two approaches, the Sullivan technique [32] was employed. Using the Sullivan technique, the DFLE at age *x* was calculated using the following formula:

$${DFLE}_{x}= \frac{1}{{l}_{x}}\sum_{a=x}^{\omega }{L}_{a}{\pi }_{a}$$

where, ${l}_{x}$ indicates to the number of survivors at age $x$; ${L}_{a}$ means person-years lived for the age interval $a$, and ${\pi }_{a}$ refers to the prevalence of disability-free for the age interval $a$.

## Results

The overall and age-specific participation of males and females in the study of urban–rural differences in disability-free life expectancy in Bangladesh 2016 is presented in Table 1. This study was conducted using the data of HIES-2016, where the total respondents were 186,055 among them 49.73% were male and 50.27% were female. The ages were divided into different groups. The participation of age the group 10–14 years was very high (11.46%) while the age group 80 + years was very low (0.9%) compared to others. The eyesight difficulties were reported by 4.16%. Additionally, hearing difficulties, walking difficulties, concentration difficulties, self-care difficulties, and communication difficulties were reported by 2.07%, 2,03%, 1.51%, 1.35%, and 1.28% respectively (Table 1).

**Table 1:** Descriptive analysis of the study subjects

Characteristics	Frequency	Percentage
*Gender*		
Male	92,527	49.73
Female	93,528	50.27
*Age group (years)*		
< 1	2,852	1.53
1–4	15,108	8.12
5–9	19,699	10.59
10–14	21,334	11.46
15–19	18,091	9.72
20–24	14,471	7.78
25–29	16,164	8.69
30–34	14,174	7.62
35–39	13,889	7.46
40–44	10,546	5.67
45–49	10,206	5.48
50–54	7,760	4.17
55–59	6,492	3.49
60–64	5,547	2.98
65–69	3,942	2.12
70–74	2,843	1.53
75–79	1,287	0.69
80 +	1,681	0.9
*Difficulty in seeing, even if he or she wears glasses?*		
No difficulty	178,305	95.84
Yes, some difficulty	6,923	3.72
Yes, severe difficulty	661	0.36
Yes, can’t see/ hear/ walk/ remember/ self-care/ communicate at all	157	0.08
*Difficulty in hearing, even if he or she is wearing a hearing aid?*		
No difficulty	182,201	97.93
Yes, some difficulty	3,220	1.73
Yes, severe difficulty	457	0.25
Yes, can’t see/ hear/ walk/ remember/ self-care/ communicate at all	170	0.09
*Difficulty in walking/climbing or any other physical movement?*		
No difficulty	182,272	97.97
Yes, some difficulty	2,674	1.44
Yes, severe difficulty	808	0.43
Yes, can’t see/ hear/ walk/ remember/ selfcare/ communicate at all	293	0.16
*Difficulty in remembering or concentrating?*		
No difficulty	183,243	98.49
Yes, some difficulty	1,957	1.05
Yes, severe difficulty	559	0.3
Yes, can’t see/ hear/ walk/ remember/ selfcare/ communicate at all	287	0.15
*Difficulty in (with self-care such as) washing all over, dressing, feeding, toileting etc.?*		
No difficulty	183,507	98.64
Yes, some difficulty	1,562	0.84
Yes, severe difficulty	552	0.3
Yes, can’t see/ hear/ walk/ remember/ selfcare/ communicate at all	422	0.23
*Difficulty in communicating, that is understanding or being understood?*		
No difficulty	183,648	98.72
Yes, some difficulty	1,421	0.76
Yes, severe difficulty	521	0.28
Yes, can’t see/ hear/ walk/ remember/ selfcare/ communicate at all	436	0.23

Table 2 represents the age-specific mortality rate for the age group 80 + years was the highest for rural males compared to urban males and rural males-females both whereas, the mortality rate for urban females was the highest among rural females and urban males for the age-group 80 + years. For the age group 55+ years, the mortality rate for the rural male population was highest compared to all groups. Few exceptions were noticed for the age groups 10–14 and 25–29 years where rural females had the highest mortality rate. Aside from that, age-group 40–44 and 50–54 years, had the highest mortality rate for urban males. The last row of the table named “total” is the age-standardized mortality rate and age-standardized prevalence. The age-standardized mortality rate for the rural male population was highest (6.5 per 1000) while the age-standardized disability prevalence was highest (6.17%) for the rural female population (Table 2).

**Table 2:** Urban and rural area-based age-sex-specific mortality rate (per 1000) and disability prevalence (percent) in Bangladesh, 2016

Age group (year)	Age-sex specific mortality rate (per 1000)				Age-sex specific disability prevalence (percent)					
	Male		Female		Male			Female		
	Rural	Urban	Rural	Urban	Rural	Urban	$p$-values*	Rural	Urban	$p$-values*
< 1	33.3	34.86	34.04	18.03	5.28	6.04	0.566	6.31	6.03	0.844
1–4	2.78	1.11	1.89	1.12	7.16	7.69	0.429	7.22	6.56	0.309
5–9	0.97	0.28	0.53	0.71	4.81	4.83	0.971	4.83	4.14	0.147
10–14	0.61	0.3	0.69	0.08	2.33	2.56	0.478	2.1	1.51	0.054
15–19	1.6	1	1.44	1.04	2.17	1.85	0.319	2.2	2.16	0.916
20–24	1.07	0.58	0.8	0.54	1.82	1.82	0.999	1.98	1.75	0.480
25–29	1.1	0.56	1.26	1.21	2.34	2.2	0.714	2.59	2.89	0.407
30–34	1.29	1.13	0.95	0.95	2.74	2.8	0.875	3.09	2.23	0.038
35–39	2.61	1.51	1.97	1.2	3.58	2.41	0.010	5.51	4.41	0.054
40–44	2.84	2.93	2.63	2.37	5.38	4.52	0.179	7.78	7.21	0.475
45–49	5.13	3.15	3.7	2.82	7.16	5.21	0.008	11.03	8.86	0.018
50–54	8.71	8.86	6.2	5.87	8.82	9.74	0.344	15.24	12.83	0.059
55–59	14.84	11.05	8.49	8.49	12.52	12.97	0.718	18.38	15.52	0.060
60–64	19.04	16.89	15.98	13.81	17.12	18.32	0.447	24.86	19.51	0.003
65–69	29.23	24.73	24.63	19	22.82	22.24	0.789	28.5	24.85	0.120
70–74	44.97	39.16	40.68	35.71	31.38	25.55	0.027	36.73	34.8	0.540
75–79	64.78	57.47	43.5	43.06	39.77	20.24	0.000	46.22	42.28	0.404
80 +	112.32	111.37	104.58	111.43	48.04	43.56	0.246	54.17	47.16	0.070
Total^^^	6.5	4.7	5.0	3.7	6.4	5.69	0.000	7.7	6.17	0.000

*$p$ values represent significance of the percent difference of age-sex specific disability prevalence between urban and rural areas

The age-sex-specific disability prevalence for the rural female population was highest compared to urban female and male (urban and rural) populations belonging to the age group 30 + years. For the age groups 1–4, 5–9, and 10–14 years, the disability prevalence was found the highest for the male who lived in urban. The disability prevalence was found highest for the remaining age group 15–19, 20–24, and 25–29 years for rural males, rural females and urban females respectively. The statistical differences were noticed for only a few age-group. For the male population, the age groups 35–39, 45–49, and 75–79 years showed significant statistical differences based on the area (urban and rural). For the female population, the age groups 10–14, 30–34, 35–39, 45–49, 50–54, and 60–64 years showed significant statistical differences based on both urban and rural areas [Table 2].

**Fig. 1:**
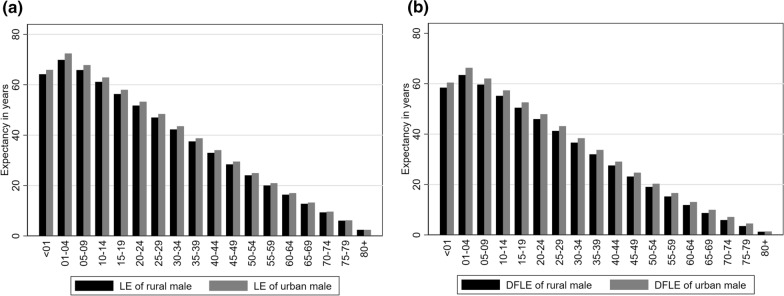
**a** Differences in life expectancy (LE) for urban and rural area male in Bangladesh, 2016 [left panel]; **b** Differences in disability-free life expectancy (DFLE) for urban and rural area male in Bangladesh, 2016 [right panel]

Table 3 presents LE, DFLE with 95% confidence interval, the proportion of expected life without disability based on area, and urban–rural differences in LE and DFLE for Bangladeshi males in 2016. Urban males aged 1–4 years have the highest LE of 72.40 years and DFLE of 66.31 years, with a difference of 2.54 years compared to rural males. The differences in LE and DFLE for urban and rural males are positive, indicating that urban males have higher LE and longer DFLE than rural males. Figure 1a and b show that the life expectancy and disability-free life expectancy both were longer for the urban male than the rural male population in any age group. Differences in disability-free life expectancy of the male population categorized into the urban and rural areas were found statistically significant from age group < 1 to age group 35–39 years. The proportion of life without disability for urban males was also found highest for the age group 1–4 years compared to rural males and all remaining age groups (Table 3).

**Table 3:** Differences in life expectancy (LE), disability-free life expectancy (DFLE), and proportion of expected life without disability for urban and rural area male in Bangladesh, 2016

Age group (year)	Life Expectancy (LE)			Disability Free Life Expectancy (DFLE)						Proportion of life without disability	
	Urban	Rural	Differences in LE* (Urban–Rural)	Urban	95% CI	Rural	95% CI	Differences in DFLE^ (Urban–Rural)	p- value°	Urban	Rural
< 1	65.89	64.16	1.73	60.45	59.94–60.96	58.43	57.78–59.08	2.02	0.05	91.73	91.07
1–4	72.40	69.87	2.54	66.31	65.80–66.82	63.44	62.79–64.09	2.86	0.01	91.58	90.81
5–9	67.80	65.82	1.98	62.05	61.54–62.56	59.67	59.02–60.32	2.39	0.02	91.53	90.65
10–14	62.89	61.13	1.76	57.38	56.87–57.89	55.19	54.53–55.85	2.19	0.02	91.24	90.28
15–19	57.98	56.31	1.67	52.59	52.07–53.11	50.46	49.80–51.12	2.12	0.05	90.70	89.62
20–24	53.26	51.74	1.52	47.93	47.41–48.45	45.96	45.30–46.62	1.97	0.05	90.00	88.82
25–29	48.41	47.00	1.40	43.16	42.64–43.68	41.28	40.62–41.94	1.87	0.05	89.15	87.83
30–34	43.53	42.25	1.28	38.38	37.85–38.91	36.61	35.94–37.28	1.77	0.05	88.16	86.66
35–39	38.77	37.51	1.26	33.72	33.19–34.25	31.97	31.30–32.64	1.75	0.05	86.99	85.24
40–44	34.04	32.97	1.08	29.08	28.54–29.62	27.54	26.86–28.22	1.54	0.1	85.43	83.53
45–49	29.50	28.40	1.11	24.70	24.16–25.24	23.16	22.48–23.84	1.53	0.1	83.71	81.57
50–54	24.93	24.07	0.86	20.31	19.77–20.85	19.06	18.38–19.74	1.25	0.2	81.47	79.19
55–59	20.94	20.02	0.92	16.61	16.06–17.16	15.24	14.56–15.92	1.37	0.2	79.32	76.13
60–64	16.98	16.36	0.62	13.07	12.52–13.62	11.87	11.18–12.56	1.20	0.2	76.98	72.52
65–69	13.25	12.74	0.51	9.95	9.40–10.50	8.69	8.00–9.38	1.26	0.2	75.12	68.24
70–74	9.65	9.34	0.31	7.11	6.56–7.66	5.89	5.19–6.59	1.22	0.2	73.66	63.05
75–79	6.19	6.06	0.13	4.52	3.96–5.08	3.50	2.80–4.20	1.01	0.2	72.94	57.85
80 +	2.42	2.42	0.00	1.37	0.81–1.93	1.26	0.55–1.97	0.11	> 0.2	56.44	51.96

CI = confidence interval of DFLE, *Due to a paucity of data, LE differences were not statistically tested, ^DFLE differences were statistically tested with $Z$-statistic, °$p$-values provided significance for a two-tailed test for DFLE differences between urban and rural areas

**Fig. 2:**
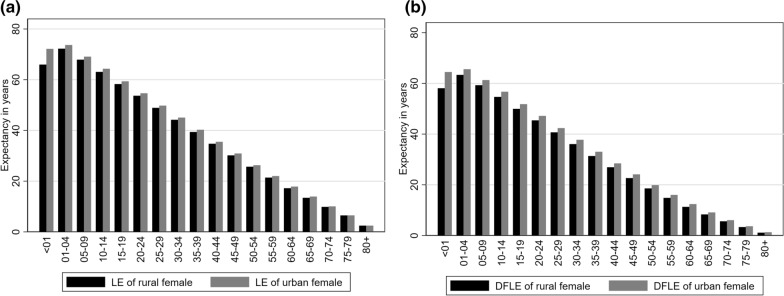
**a** Differences in life expectancy (LE) for urban and rural area female in Bangladesh, 2016 [left panel]; **b** Differences in disability-free life expectancy (DFLE) for urban and rural area female in Bangladesh, 2016 [right panel]

Table 4 displays the life expectancy (LE), disability-free life expectancy (DFLE) with a 95% confidence interval, and proportion of expected life without disability based on rural and urban areas, as well as urban–rural differences in LE and DFLE for all age groups of Bangladeshi females in 2016. Rural females in the 1–4 age group have a longer life expectancy at 73.68 years compared to rural females in other age groups. Positive values across all age groups indicate that urban females have a longer life expectancy and disability-free life expectancy than rural females. Additionally, rural females in the 1–4 age group have a longer disability-free life expectancy at 63.33 years compared to rural females in other age groups. Figure 2a and b show that the life expectancy and disability-free life expectancy were longer for the urban female than the rural female population in any age group. Differences in disability-free life expectancy of the female population categorized into the urban and rural areas were also found statistically significant from age group < 1 to age group 35–39 years. The proportion of life without disability for urban females was also found highest for the age group < 1 compared to rural males and all remaining age groups.

**Table 4:** Differences in life expectancy (LE), disability-free life expectancy (DFLE), and proportion of expected life without disability for urban and rural area female in Bangladesh, 2016

Age group (year)	Life Expectancy (LE)			Disability Free Life Expectancy (DFLE)						Proportion of life without disability	
	Urban	Rural	Differences in LE* (Urban–Rural)	Urban	95% CI	Rural	95% CI	Differences in DFLE^ (Urban–Rural)	p- value°	Urban	Rural
< 1	72.17	65.97	6.20	64.48	63.95–65.01	58.10	57.43–58.77	6.38	< 0.01	89.35	88.07
1–4	73.68	72.22	1.45	65.59	65.07–66.11	63.33	62.65–64.00	2.27	0.01	89.03	87.68
5–9	69.08	67.89	1.19	61.28	60.76–61.80	59.28	58.60–59.95	2.01	0.02	88.71	87.31
10–14	64.32	63.07	1.25	56.70	56.17–57.23	54.67	53.99–55.35	2.03	0.02	88.15	86.68
15–19	59.34	58.28	1.06	51.80	51.27–52.33	49.95	49.27–50.64	1.84	0.05	87.28	85.72
20–24	54.64	53.68	0.96	47.16	46.63–47.69	45.41	44.72–46.09	1.75	0.05	86.31	84.59
25–29	49.78	48.88	0.89	42.37	41.85–42.89	40.68	39.99–41.36	1.69	0.05	85.11	83.21
30–34	45.06	44.18	0.89	37.75	37.21–38.29	36.05	35.35–36.74	1.71	0.05	83.78	81.60
35–39	40.27	39.37	0.89	33.03	32.49–33.57	31.36	30.67–32.05	1.67	0.05	82.04	79.65
40–44	35.49	34.74	0.75	28.44	27.90–28.98	26.92	26.22–27.63	1.51	0.1	80.12	77.50
45–49	30.88	30.16	0.72	24.11	23.56–24.66	22.64	21.93–23.34	1.47	0.1	78.06	75.04
50–54	26.29	25.68	0.61	19.86	19.32–20.40	18.57	17.86–19.27	1.29	0.1	75.55	72.31
55–59	21.99	21.40	0.59	16.02	15.47–16.57	14.84	14.14–15.55	1.18	0.1	72.87	69.35
60–64	17.83	17.22	0.61	12.40	11.84–12.96	11.31	10.60–12.02	1.09	0.2	69.53	65.70
65–69	13.91	13.43	0.49	9.11	8.55–9.67	8.33	7.62–9.04	0.78	> 0.2	65.45	62.03
70–74	10.04	9.85	0.19	6.06	5.51–6.61	5.60	4.88–6.32	0.46	> 0.2	60.35	56.88
75–79	6.50	6.49	0.01	3.65	3.08–4.22	3.33	2.61–4.06	0.32	> 0.2	56.24	51.37
80 +	2.45	2.45	0.00	1.29	0.72–1.86	1.12	0.39–1.86	0.17	> 0.2	52.84	45.83

CI = confidence interval of DFLE, *Due to a paucity of data, LE differences were not statistically tested, ^DFLE differences were statistically tested with $Z$-statistic, °$p$-values provided significance for a two-tailed test for DFLE differences between urban and rural areas

## Discussion

This study reports a disability prevalence of 6.72% based on HIES 2016 data, showcasing gender and rural–urban variations (5.72% in males, 7.24% in females, 7.06% in rural, and 5.93% in urban). In contrast, The National Survey on Persons with Disabilities (NSPD) 2021 [5] in Bangladesh reveals a lower prevalence of 2.80%, with gender and geographical disparities (3.28% in males, 2.32% in females, 2.89% in rural, and 2.45% in urban areas). The difference in disability percentages between NSPD 2021 and this study, based on HIES 2016, likely arises from divergent definitions of disability inclusion. NSPD 2021, aligning with the Persons with Disability Rights and Protection Act, 2013 of Bangladesh, broadly defines disability as long-term impairments impacting societal participation. On the contrary, this study categorized individuals as “disabled” based on HIES-2016 criteria, which utilized the Washington Group framework and ICF principles. Disability was determined if individuals reported difficulty in vision, hearing, mobility, memory/concentration, self-care, or communication, with varying severity levels (some, severe, or complete inability) in any of these areas. Detailed methodology regarding disability selection is outlined in the methodology section of this study. A valuable picture of disability prevalence in Bangladesh is portrayed by our study, focusing on six functional limitations. However, a far richer tapestry is revealed by the NSPD 2021, encompassing diverse impairments and highlighting stark disparities in access to essential healthcare services and insurance for people with disabilities. Higher disability rates than suggested by our study are unveiled by NSPD data, and rural–urban divides mirror our findings of higher mortality in rural areas, particularly among certain urban male age groups. A multifaceted approach to policy and program development is demanded by this intricate interconnectedness.

Beyond disability-specific interventions, insurance coverage expansion is proposed, as advocated by the NSPD 2021, to address the disparities identified by both studies. Specific programs should cater to rural populations struggling with higher mortality rates and the diverse needs of individuals with varying disabilities. Rural healthcare infrastructure and service delivery must be strengthened to match urban standards. Adopting the NSPD 2021’s holistic approach, incorporating the ICF framework, will ensure a complete understanding of disability’s impact on individuals and society. Tackling attitudinal and environmental barriers, as the NSPD 2021 emphasizes, is critical for full social inclusion and participation for people with disabilities. A richer, more informed tapestry of healthcare access and disability experiences in Bangladesh can be created by weaving together findings from our study, the NSPD 2019 [6], and the NSPD 2021. Only then can truly inclusive policies and programs be crafted that address the intricate needs of all individuals, regardless of location, disability, or other circumstances, paving the way for a future where health and well-being are woven into the fabric of every life.

The age-sex-specific disability prevalence indicates significant differences among urban and rural populations, influenced by factors like limited healthcare access, emergency response times, and socioeconomic status [15]. Women in urban areas have lower mortality rates than those in rural areas, except for the 5–9 and 80 + years age groups. The difference in mortality rates across gender and location is attributed to various factors, which will be discussed later. Among urban and rural males, there is a significant difference in age-sex-specific disability prevalence for the 35–39, 45–49, 70–74, and 75–79-year age groups. Rural males have higher disability prevalence than urban males in these age groups. Similarly, rural females have higher disability prevalence in the 10–14, 30–34, 45–49, and 60–64-year age groups. The findings indicate disability prevalence increases significantly with age, which is consistent with a 2018 study [26].

The results indicate that rural females have significantly higher age-sex-specific disability prevalence than urban females, except for a few age groups. This difference may be attributed to several factors, including limited access to maternal and infant healthcare services, longer emergency response times, poverty, and a shortage of physicians and nurses in rural areas (UNFPA 2019). Socio-economic status and access to healthcare services may be risk factors for higher mortality rates in rural areas, as well as the lack of education and knowledge about healthcare facilities. These factors may also contribute to higher disability rates among rural populations. Past studies have suggested that social disadvantage is a major contributing factor to disability [13, 22]. This urban and rural mortality scenario goes with the worldwide urban–rural difference in mortality and many aspects [38]. Urban males have a higher life expectancy than rural males across all age groups, with differences ranging from 0 to 2.54. Additionally, urban males have a higher disability-free life expectancy than rural males, with differences ranging from 0.11 to 2.86, particularly for the age group 0 to 39. Urban females also outperform rural peers in life expectancy, disability-free life expectancy, and a higher proportion living without disabilities, particularly in the 0 to 39 age group. Conversely, rural males and females exhibit lower life expectancy and disability-free life expectancy, with a smaller population living without disabilities. Infants in rural areas face significantly lower life expectancy, underscoring the need for increased attention to rural females under one year old. Additionally, rural females live longer with disabilities compared to urban counterparts.

This study gives a spotlight on the significant difference in age-sex-specific mortality, life expectancy, and disability-free life expectancy in urban and rural areas, especially among people at age < 1–40 years. Similar findings come out in a study in neighboring country India, higher rate of age-sex-specific mortality and life expectancy without disability in rural areas [30]. The study also found that Bangladeshi women expect a smaller proportion of life without disability compared with men, which is also in line with findings that women spend less time in disabled states than men [7, 10, 12, 27, 31].

## Limitations

The fact that the data included in the study was self-reported is one of the study’s disadvantages. Although this could be a source of bias, a study [42] discovered that self-reported functional impairment data were consistent with medical diagnosis. The second issue is that proxy interviews for older persons who were unable to participate in the interviews were not mentioned in the HIES 2016 survey. Then, the institutionalized population was not considered due to a lack of data. If the institutionalized population is not taken into consideration, the DFLE may be overestimated, especially as people get older [39]. The Sullivan method, employed for estimating Disability-Free Life Expectancy (DFLE), has notable limitations [14]. Firstly, its reliance on cross-sectional data provides a static view, potentially overlooking transitions between health and disability states and leading to inaccurate DFLE estimates. Secondly, the assumption of constant disability prevalence across age groups simplifies reality, potentially underestimating age-related increases in disability and affecting DFLE estimates. Lastly, the method may overlook temporary impairments, fluctuations in disability severity, or potential recovery, thus limiting its accuracy in capturing the dynamic nature of disability. These limitations warrant careful consideration when utilizing the Sullivan method in studies focusing on DFLE estimation. Detailed life tables that consider education, marital status, religion, and residence are needed to better understand disability causes and solutions. This study assumes that institutionalized elderly people have similar health problems and disabilities as the general population. Despite these limitations, recent nationally representative statistics for older people in Bangladesh show significant gender disparities in disability and DFLE.

## Conclusion

This study comprehensively analyzed disability-free life expectancy (DFLE) in urban and rural areas of Bangladesh using HIES 2016 and SVRS 2016 data. Significant disparities were uncovered in disability prevalence, mortality rates, life expectancy (LE), and DFLE across genders and geographic locations. The most prevalent disability was found to be the difficulty in seeing, which can be mitigated with interventions like providing spectacles and enhancing cataract surgical treatments. Targeted interventions and policy adjustments are imperative, particularly in rural areas, to address the identified disparities. Proper care and the implementation of focused projects emerged as crucial factors for reducing disability prevalence. Despite higher mortality and disability rates in rural areas, urban males and females consistently exhibited longer LE, DFLE, and a higher proportion of life without disability, particularly in the age group 0 to 39. The findings underscore the urgency for addressing health disparities through targeted interventions and policy adjustments, particularly in rural regions. Recognizing the study’s insights into urban–rural and gender-specific patterns of LE and DFLE, appropriate measures can be taken to narrow disparities in economic development, healthcare access, education, and infrastructure, ultimately contributing to achieving urban–rural equality in LE and DFLE. Addressing socioeconomic factors, such as infrastructure development and accessible healthcare, is crucial for informing health policy in Bangladesh.

## Data Availability

The national Household Income Expenditure Survey (HIES) is conducted by Bangladesh Bureau of Statistics (BBS) with technical and financial assistance from the World Bank. The data is available publicly for purchase from the Bangladesh Bureau of Statistics. The datasets analyzed during the current study are available in the Bangladesh Bureau of Statistics (BBS) Data Catalog Application repository through the link, http://data.bbs.gov.bd/index.php/catalog/182#:~:text=Household%20Income%20and%20Expenditure%20Survey%20%28HIES%29%20is%20one,in%20the%20decision%20making%20process%20for%20the%20government.
